# Comparing spatial distributions of ALA-PpIX and indocyanine green in a whole pig brain glioma model using 3D fluorescence cryotomography

**DOI:** 10.1117/1.JBO.30.S1.S13704

**Published:** 2024-09-06

**Authors:** Augustino V. Scorzo, Caleb Y. Kwon, Rendall R. Strawbridge, Ryan B. Duke, Kristen L. Chen, Chengpei Li, Xiaoyao Fan, P. Jack Hoopes, David W. Roberts, Keith D. Paulsen, Scott C. Davis

**Affiliations:** a Dartmouth College, Thayer School of Engineering, Hanover, New Hampshire, United States; b Dartmouth College, Geisel School of Medicine, Hanover, New Hampshire, United States; c Norris Cotton Cancer Center, Dartmouth-Hitchcock Medical Center, Lebanon, New Hampshire, United States

**Keywords:** fluorescence cryotomography, fluorescence-guided surgery, ALA-PpIX, indocyanine green, Oncopig

## Abstract

**Significance:**

ALA-PpIX and second-window indocyanine green (ICG) have been studied widely for guiding the resection of high-grade gliomas. These agents have different mechanisms of action and uptake characteristics, which can affect their performance as surgical guidance agents. Elucidating these differences in animal models that approach the size and anatomy of the human brain would help guide the use of these agents. Herein, we report on the use of a new pig glioma model and fluorescence cryotomography to evaluate the 3D distributions of both agents throughout the whole brain.

**Aim:**

We aim to assess and compare the 3D spatial distributions of ALA-PpIX and second-window ICG in a glioma-bearing pig brain using fluorescence cryotomography.

**Approach:**

A glioma was induced in the brain of a transgenic Oncopig via adeno-associated virus delivery of Cre-recombinase plasmids. After tumor induction, the pro-drug 5-ALA and ICG were administered to the animal 3 and 24 h prior to brain harvest, respectively. The harvested brain was imaged using fluorescence cryotomography. The fluorescence distributions of both agents were evaluated in 3D in the whole brain using various spatial distribution and contrast performance metrics.

**Results:**

Significant differences in the spatial distributions of both agents were observed. Indocyanine green accumulated within the tumor core, whereas ALA-PpIX appeared more toward the tumor periphery. Both ALA-PpIX and second-window ICG provided elevated tumor-to-background contrast (13 and 23, respectively).

**Conclusions:**

This study is the first to demonstrate the use of a new glioma model and large-specimen fluorescence cryotomography to evaluate and compare imaging agent distribution at high resolution in 3D.

## 1 Introduction

The use of ALA-PpIX fluorescence to guide the removal of high-grade glioma is one of the most widely adopted applications of fluorescence-guided surgery (FGS). This procedure involves administering the prodrug 5-aminolevulinic acid (5-ALA) to patients, which results in the accumulation of the endogenous fluorophore protoporphyrin IX (PpIX) in neoplastic cells enabling fluorescence guidance.^1^^–^^3^ Despite providing a proven benefit in progression-free survival,^3^ ALA-PpIX has shown relatively low negative predictive values.^4^^,^^5^ Efforts to identify alternative or complementary fluorescent agents that could help improve clinical outcomes across brain tumor subtypes are ongoing.^6^^–^^12^ One approach that has undergone extensive preclinical and clinical evaluation over the past decade involves administering indocyanine green (ICG), a commonly used fluorophore approved for vascular visualization during angiography, 24 h or longer prior to surgery and using the fluorescence retained in the tumor at this long time point for surgical guidance.^7^^,^^8^^,^^13^^–^^15^ This technique, often referred to as “second-window” ICG (SWIG) imaging, has demonstrated high sensitivity and negative predictive values in detecting neoplastic tissue in many cases (>90% and >70%, respectively).^14^^–^^17^ In this context, understanding the differences in uptake and diagnostic performance of ALA-PpIX and SWIG has emerged as an area of interest in the field. In a prior study, Cho et al.^18^ compared the two agents in xenograft mouse models and clinical tumors, finding that the agents were generally correlated except in necrotic regions. Although this study was based on analysis of 2D images, examination of the full volumetric distribution throughout the brain of a large animal model may reveal further insights into the behavior of these two agents.

In this study, we compared the distribution of ALA-PpIX and SWIG fluorescence in three dimensions in a transgenic swine glioma model. Specifically, we induced an endogenous tumor in the brain of an oncogenic pig model using Cre-recombinase-expressing adeno-associated viral (AAV) vectors. After a tumor was identified using contrast-enhanced magnetic resonance imaging (MRI) monitoring, SWIG and 5-ALA were administered to the animal, and at a pre-defined assessment time, the whole brain was harvested and imaged using fluorescence cryo-imaging. The cryo-imaging process sectioned and imaged the whole brain specimen to produce high-resolution 3D volumes of both agents, perfectly registered to one another, throughout the tumor and normal brain. Agent distribution and contrast metrics were compared using tumor profiles, mean intensity from the tumor boundary region, tumor-to-background ratio (TBR), contrast-to-noise ratio (CNR), normalized cross-correlation (CC), and joint histogram analysis.

## 2 Experimental Design

**Fig. 1:**
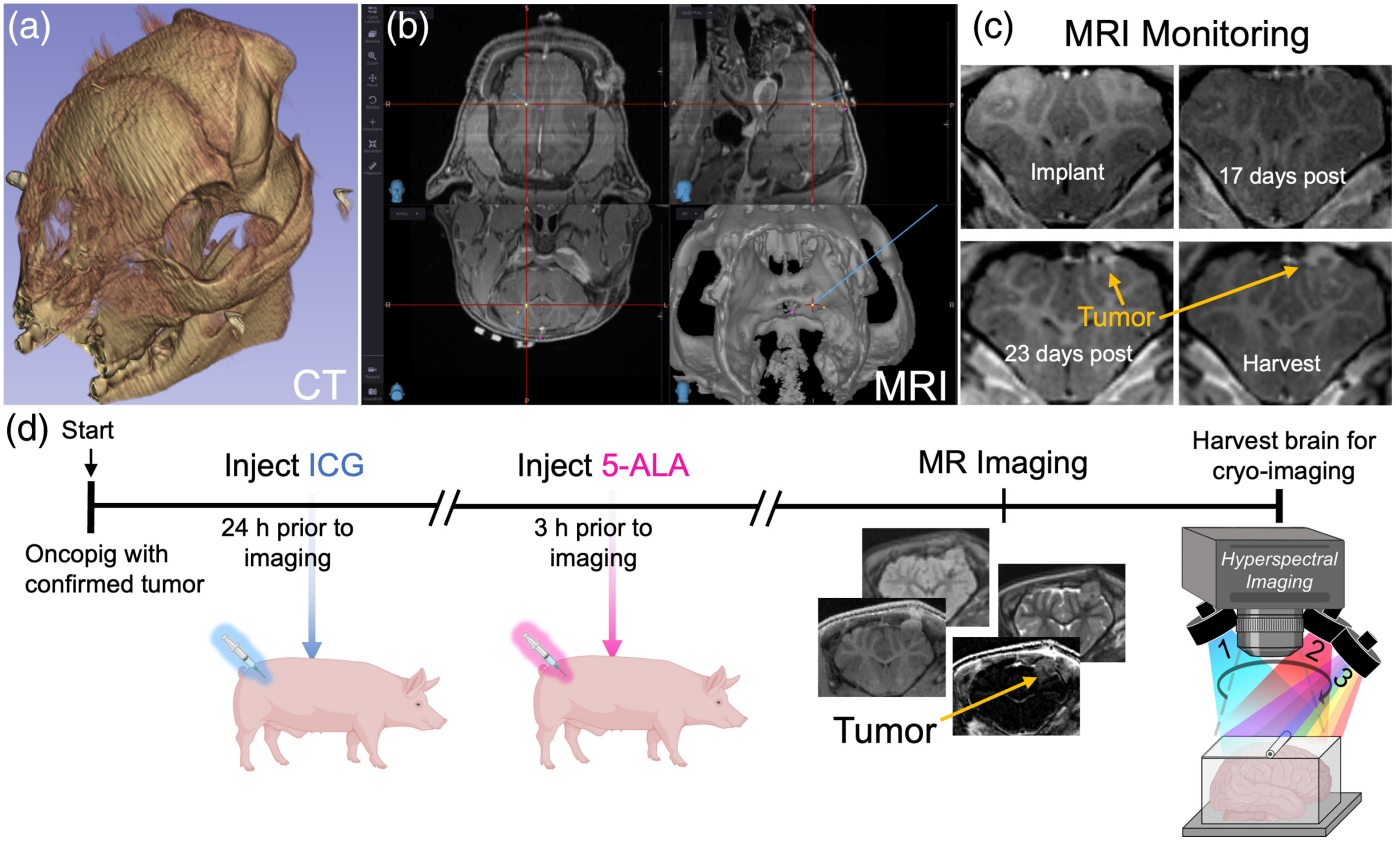
3D rendering of the pig skull from CT scans (a) and co-registered MRI scans (b) were used to triangulate the intracranial implantation of AAV. (c) Representative post-contrast MRI images during tumor growth monitoring. (d) Experimental timeline for contrast agent administration and whole brain specimen imaging.

All procedures were conducted in accordance with protocols approved by the Institutional Animal Care and Use Committee at Dartmouth College. The animal used in this study was a 10-week-old female transgenic Oncopig (NSRRC:0033) obtained from the National Swine Resource and Research Center (Columbia, Missouri, United States).^19^ The DNA in this model encodes the mutated oncogene and tumor suppressor genes KRAS^G12D^ and TP53^R167H^, respectively, with an adjacent lox-STOP-lox cassette. These genes are not expressed until the STOP cassette is excised by the Cre-recombinase enzyme. Local intracranial administration of an AAV carrying a Cre-recombinase plasmid can induce the expression of the mutated genes in infected cells, which in turn drives local carcinogenesis. To initiate this process, the Oncopig was anesthetized and imaged using intraoperative computed tomography (CT) and 3T intraoperative MRI scanners to plan AAV implantation. A hole was then drilled into the skull ∼4  mm left and 10 mm cranial of bregma, and a needle was positioned 10 mm into the brain under CT and MRI guidsaance, as illustrated in Figs. 1(a) and 1(b). A 10  μL cocktail of AAVs with four different promoters was administered into the brain, namely, pENN.AAV.hSyn.Cre.WPRE.hGH (Addgene 105535-AAV5, Watertown, Massachusetts, United States), pENN.AAV.CMVs.Pl.Cre.rBG (Addgene 105537-AAV2), pENN.AAV.CMVs.Pl.Cre.rBG (Addgene 105537-AAV9), and AAV-GFAP(2.2)-iCre (Vector Biolabs VB1172, Malvern, Pennsylvania, United States). Tumor growth was monitored using contrast-enhanced T1-weighted MRI volumes approximately every 7 to 14 days [Fig. 1(c)]. After 33 days, MRI enhancement indicated the presence of a $208\;{\mathrm{mm}}^{3}$ tumor, and the animal was put on study.

A schematic of the experimental timeline showing the administration and imaging times is provided in Fig. 1(d). A 5  mg/kg human-equivalent dose of ICG (MWI Animal Health, Conshohocken, Pennsylvania, United States) was dissolved in sterile water prior to use and administered through an indwelling catheter in the lateral auricular vein 24 h prior to euthanasia. Three hours prior to euthanasia, a 20  mg/kg human-equivalent dose of 5-ALA (Thermo Fisher Scientific, Waltham, Massachusetts, United States) was delivered via the same catheter. At the designated time after agent administration, a final set of MRI images was acquired on a Siemens Prisma 3.0T MRI scanner using a 20-channel head coil. The animal was then euthanized, the brain was harvested, and a tumor specimen was collected for pathological analysis using hematoxylin and eosin staining and anti-glial fibrillary acidic protein (GFAP) immunohistochemistry (Fig. S1 in the Supplementary Material). The whole brain was then submerged in optimal cutting temperature compound (Fisher Scientific, Pittsburgh, Pennsylvania, United States) and frozen at −20°C in preparation for fluorescence cryotomography.

The fluorescence cryotomography system has been described previously and provides image stacks of spectrally distinct fluorescent agents and white-light RGB through autonomous sectioning and imaging.^20^ In this study, the whole brain was imaged using a white light emitting diode (LED) (Mightex, Toronto, Ontario, Canada) for RGB reconstruction, a 405 nm LED (Mightex) for PpIX excitation, and a 760 nm laser (CrystaLaser LC, Reno, Nevada, United States) for ICG excitation. Remitted light from the whole-brain specimen was captured by an objective lens, split into visible and NIR channels by a 750 nm short pass dichroic mirror before additional filtering. Specifically, ICG fluorescence, directed through the NIR channel, was filtered using a 780 nm long pass filter before being focused on an sCMOS camera (Edge 4.2, PCO, Bavaria, Germany). PpIX fluorescence, directed through the visible channel, was filtered with a 460 nm long pass filter and then a liquid crystal tunable filter (LCTF, Varispec, Woburn, Massachusetts, USA) before being imaged with a second sCMOS. White light for RGB reconstruction was also directed through this channel and filtered with the LCTF only. The RGB, PpIX, and ICG image stacks were then processed and visualized using 3D Slicer.^21^^,^^22^

Tumor and normal brain volumes were identified based on contrast in the RGB image volumes, which were used to guide volume segmentation in 3D Slicer. These segmented volumes were then used to compute contrast metrics and examine the spatial distributions of the two agents. Specifically, we assessed:

*Tumor-to-background ratio (TBR)* in two ways: (1) by computing the ratio of mean fluorescence in the whole tumor region to mean fluorescence in an equivalently sized volume in the contralateral brain and (2) by computing the ratio between the mean fluorescence of the outer 1 mm volume of the tumor region and adjacent 1 mm peritumoral volume.

*Contrast-to-noise ratio (CNR)*, defined as the difference between the mean fluorescence of the tumor and normal brain divided by the standard deviation of the normal brain, was computed for the whole tumor and margin regions, as described by the two TBR methods above.

*Variation with distance from the tumor boundary region* was computed to examine the changes in mean fluorescence intensity moving away from the segmented tumor border. Specifically, starting with the RGB-volume-defined tumor boundary, image erosion was used to generate a 3D shell by moving 1 mm toward the center of the tumor. Then, the next shell was generated by starting with the inner surface of the previous shell and moving inward by 1 mm again. This process was repeated until concentric shells were created throughout the tumor volume. A similar strategy with image dilation was used to create shells from the outer tumor surface into the normal brain. Mean fluorescence values in each shell were computed for each agent.

*ALA-PpIX v. ICG joint histogram* with 100 bins was computed for normalized values of the two agents within the segmented tumor region using the hist3 bivariate histogram function in MATLAB.

*Normalized cross-correlation (CC)* between the ICG and PpIX fluorescence volumes was computed by dividing the covariance between the two agents by the product of each agent’s standard deviation. This was calculated for the bulk tumor volume and a 2 mm thick volume spanning the RGB-defined tumor boundary.

## 3 Results

**Fig. 2:**
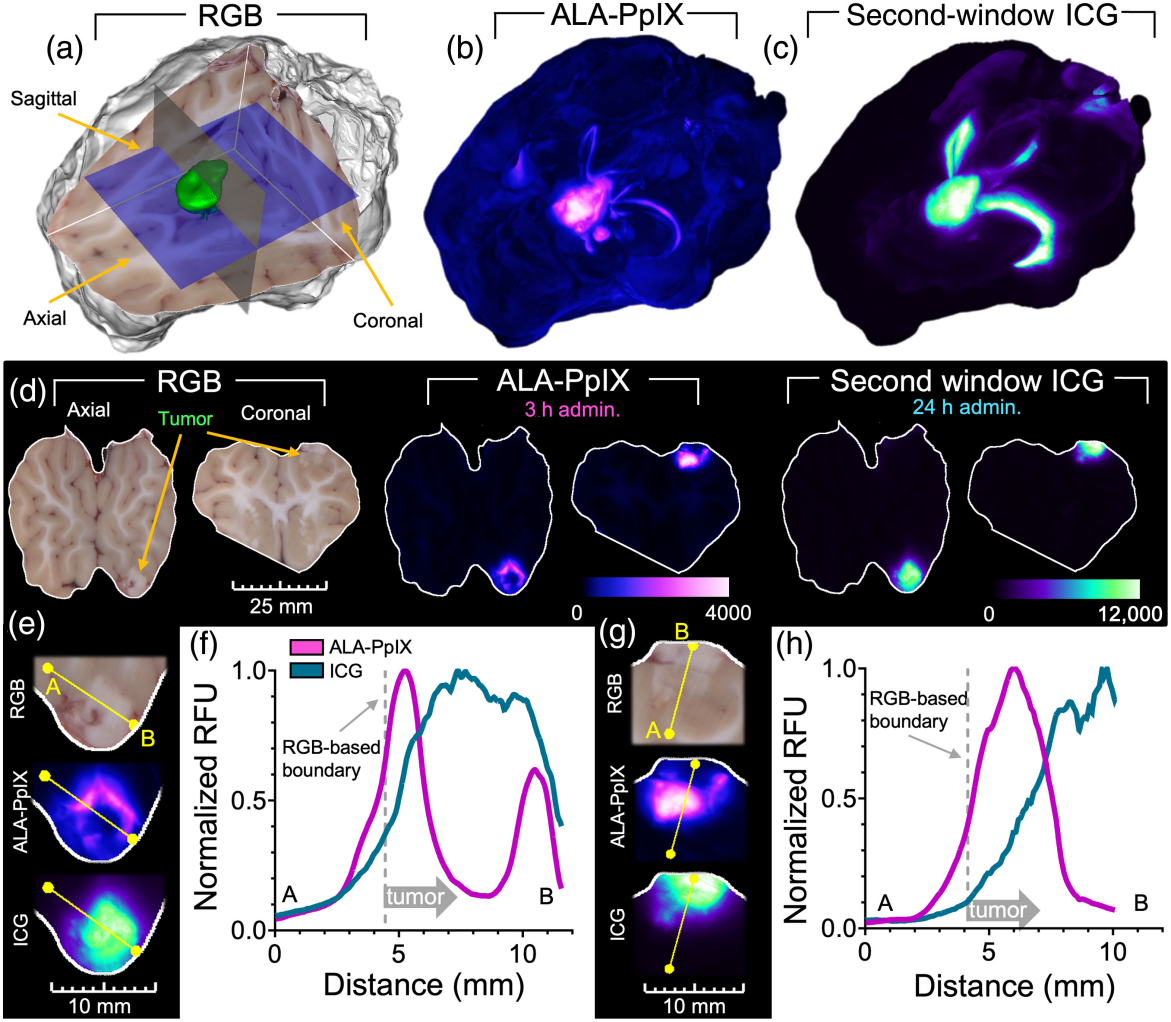
Fluorescence cryo-imaging of a whole pig brain: (a) RGB rendering with tumor surface shown in green. (b) and (c) Maximum intensity projection (MIP) images of ALA-PpIX and ICG channels, respectively. (d) 2D image slices for each channel sampled from the volume with the slice locations depicted by the two semitransparent planes shown in panel (a). (f) Normalized fluorescence intensity profiles of both agents for the line overlaid on the 2D axial slice in panel (e). Panels (g) and (h) are the same as panels (e) and (f) for the coronal slice (Video 1, MP4, 23.3 MB [URL: https://doi.org/10.1117/1.JBO.30.S1.S13704.s1]).

Figures 2(a)–2(c) present cryo-imaging volumes of the whole brain for the RGB, ALA-PpIX, and SWIG channels, respectively, and a video of these volumes is provided in Video 1. The semi-transparent planes overlaid on the RGB volume in Fig. 2(a) indicate the positions of the 2D orthogonal slices provided in Fig. 2(d). A surface rendering of the segmented tumor region appears as a green overlay in Fig. 2(a). Inspection of the fluorescence renderings and 2D planes in Fig. 2(d) indicates that both agents produced significant tumor-to-normal tissue enhancement yet starkly different spatial distributions. The ICG signal appears to be highest in the central regions of the tumor, decreasing toward the segmented tumor boundary, whereas PpIX fluorescence intensity is highest near the edges of the tumor and lower toward the tumor’s center. This behavior is further illustrated by the line profile plots shown in Figs. 2(e)–2(h), which provide cross-sections of normalized fluorescence through the tumor from axial and coronal slices measured in normalized relative fluorescence units. In these plots, the PpIX fluorescence intensity in the center of the tumor is only slightly elevated compared with normal tissue yet almost eight times higher near the tumor boundary. ICG is highest in the central region and decreases steadily toward the normal brain. In addition, both agents highlighted ventricular structures, although this was more pronounced in the ICG channel. These features were not observed in the brain of a control Oncopig not administered the contrast agents (Fig. S2 in the Supplementary Material).

**Fig. 3:**
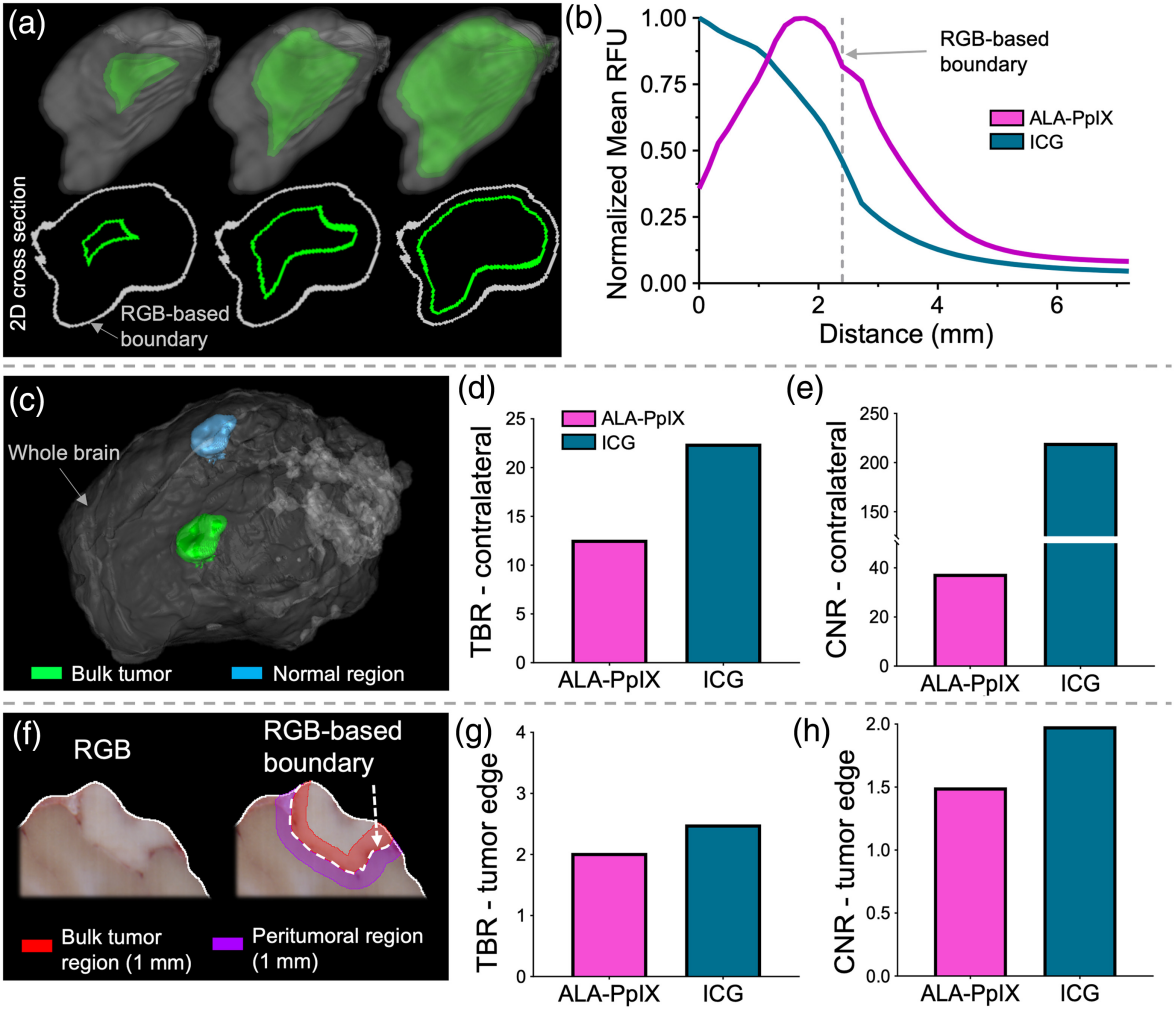
(a) Three rendered shell (in green) examples used in the concentric shell analysis, with corresponding 2D cross sections shown below. The tumor outline defined by the RGB image volume appears in gray. The volume of the walls of each shell was used to compute the mean fluorescence of each agent, which was then plotted as a function of distance from the tumor center (b). (c) Tumor and normal (contralateral) brain regions used to compute TBR and CNR shown in panels (d) and (e). (f) Single slice illustrating the regions used to compute TBR and CNR based on the tissue within 1 mm of the tumor region (note this was computed in 3D). (g)–(h) TBR and CNR using the regions within 1 mm of the tumor boundary in 3D.

The cross-sectional analysis reported in Fig. 2 is limited to a small sampling of the tissue volume. To provide a more comprehensive assessment that considers the volumetric distribution of agents, we computed the mean fluorescence intensity in concentric 1 mm thick 3D shells within and outside the tumor. Illustrative examples of three intra-tumoral shells are provided in Fig. 3(a). Mean fluorescence intensity values in each volumetric shell were computed for each channel, normalized to the highest intensity shell, and plotted in Fig. 3(b). Although not as pronounced as in the profile plots shown in Fig. 2, the trends are similar. The PpIX fluorescence intensity is highest in the tumor periphery and decreases toward the center of the tumor, whereas the reverse is observed for ICG. Notably, both agents show relatively high signals in the tissue surrounding the RGB-defined tumor region, which decreases steadily with distance into the normal tissue.

The TBR and CNR metrics for both agents were computed in two ways; namely, (1) by defining the tumor as the entire tumor volume and the normal region as an equivalently-sized volume in the contralateral brain [Fig. 3(c)], a common method for determining TBR,^9^ and (2) by defining the tumor region as a 1 mm thick shell starting from the RGB-defined tumor boundary and moving inward, and the normal region as a 1 mm thick shell starting from the RGB tumor boundary and moving outward into the normal brain [Fig. 3(f)]. As seen in Fig. 3(d), both agents report high TBR (=13 and 23 for PpIX and ICG, respectively) and CNR (=38 and 220 for PpIX and ICG, respectively) when considering the whole tumor and contralateral brain, yet these metrics decrease significantly when assessing the regions close to the tumor boundary. Here, TBR is reduced to 2.0 and 2.5 for PpIX and ICG, respectively, and CNR<2 for both agents [Figs. 3(g) and 3(h)].

**Fig. 4:**
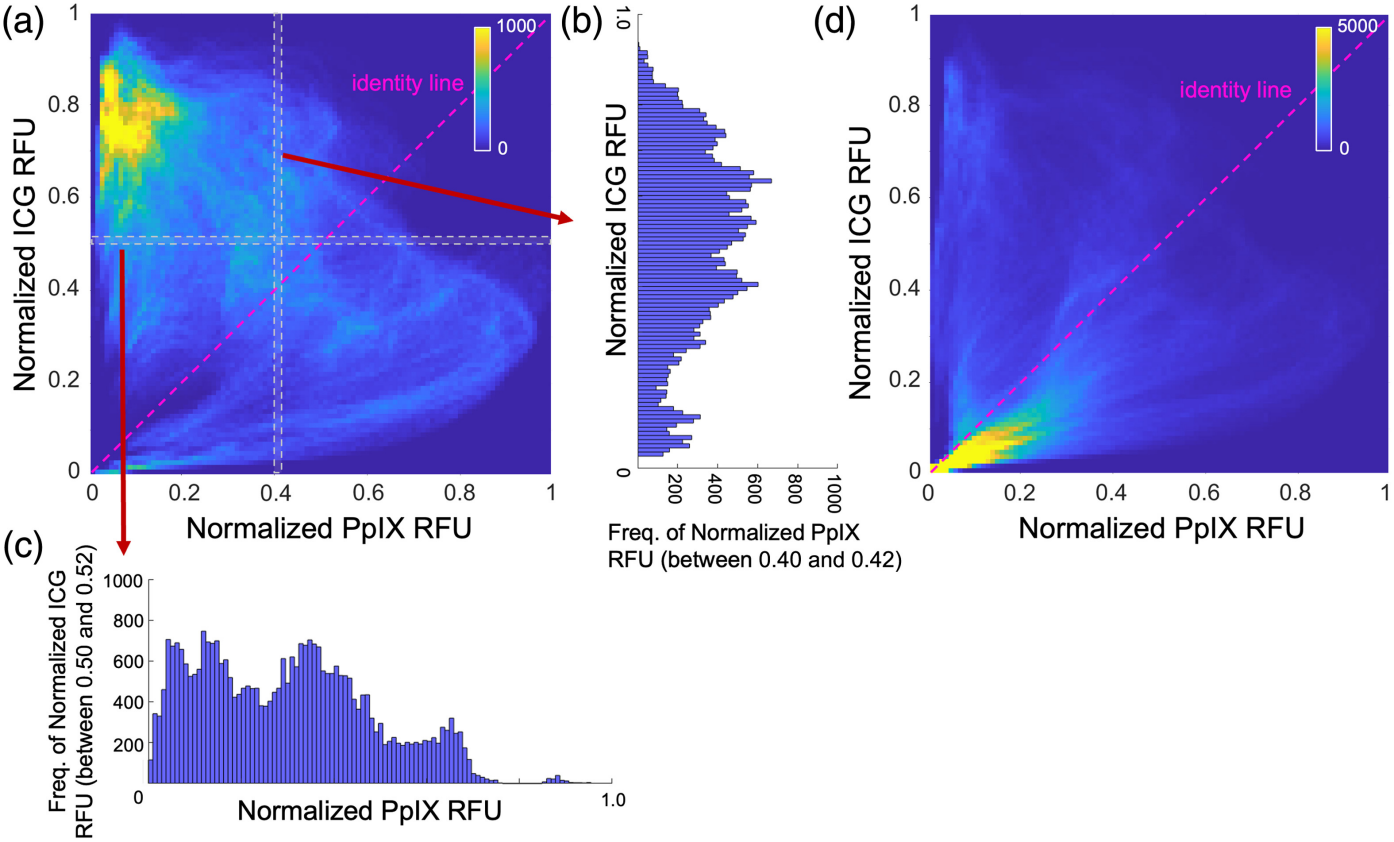
(a) Joint histogram of normalized ICG and ALA-PpIX pixel intensity values in the tumor with the frequency represented as the color bar. (b) Frequency of normalized ALA-PpIX pixel values between 0.40 and 0.42 that correspond to normalized ICG values between 0 and 1 (c) Analogous plot for the frequency of ICG pixel values between 0.50 and 0.52 corresponding to normalized ALA-PpIX values between 0 and 1. (d) Joint histogram plot of the volume within 1 mm of the RGB-defined tumor boundary.

Finally, the correlation between the two agents was examined by plotting a joint histogram and computing the cross-correlation (CC) among the volumes. Joint histograms plot the range of intensity values (histogram) from one imaging channel for a given value bin in another channel. Figure 4(a) shows the joint histogram between the ALA-PpIX and ICG channels for the entire RGB-defined tumor. The cross-sectional plots shown in Figs. 4(b) and 4(c), chosen for illustrative purposes, help elucidate the information plotted in panel (a). The x-axis in Fig. 4(b) represents ICG fluorescence intensity values (normalized), whereas the y-axis is the frequency those values appear in the volume in voxels for which ALA-PpIX has intensity values in a bin ranging between 0.4 and 0.42. Figure 4(c) is similar to Fig. 4(b) but shows the ALA-PpIX histogram for a given range of ICG values. If the two data sets were highly correlated, Fig. 4(a) would show the highest frequencies along the identity line, depicted here as a dashed pink line. This behavior is not observed here, indicating that the two data sets are not well-correlated. The calculated CC for these data was −0.47. The bright region in the top left corner of the histogram largely consists of values from the center of the tumor, in which the PpIX signal is low and the IGC signal is elevated.

Figure 4(d) is an analogous plot to Fig. 4(a) but considers the volume 1 mm to either side of the RGB-defined tumor [e.g., as illustrated by the purple and red regions in Fig. 3(f)]. In this volume, the CC was found to be 0.24, which suggests a positive though not strong correlation between the agents.

## 4 Discussion and Conclusion

To our knowledge, this study is the first to demonstrate glioma induction using the Oncopig model system, apply whole-brain fluorescence cryo-imaging in a large animal to evaluate FGS agents, and examine and compare the distribution of ALA-PpIX and ICG in 3D. Both agents provided high bulk tumor contrast with minimal normal brain signal, yet the distributions in the tumor differed considerably. Although PpIX fluorescence was observed primarily near the tumor periphery, ICG’s signal was highest in the center of the tumor. Consequently, although both agents provided high TBR and CNR (when compared with a contralateral brain), their distributions in the tumor were poorly correlated. Notably, both agents clearly highlighted the normal ventricular structures, an observation that may help address uncertainty when ventricles fluoresce in clinical cases, which was revealed by the volumetric imaging capabilities of the cryo-imaging approach.

The observed macroscopic differences between the two agents are not surprising and consistent with previous reports.^14^^,^^18^^,^^23^ ICG binds to larger protein molecules, primarily albumin, in the blood after administration, and SWIG contrast is generally thought to arise from preferential leakage and long-term retention in the tumor of the albumin-bound form of the ICG. Accumulation in the central regions of the tumor is also commonly observed with non-targeted agents after long incubation times.^18^^,^^24^^,^^25^ Conversely, the production of PpIX requires that the administered pro-drug, 5-ALA, enters cells that are metabolically active, which are typically found in the more proliferative, viable regions of the tumor that have not necrosed.^2^^,^^3^^,^^18^^,^^26^

The effect of choosing different volumes to compute TBR and CNR metrics was another notable observation. When the whole bulk tumor was compared with an equivalent contralateral brain volume, TBR>13 for both agents. Yet, when we narrowed the analysis to smaller volumes near the edge of the bulk tumor, arguably the more relevant analysis for the surgical application, the TBR dropped to around 2 for both agents. Studies to examine this question further are ongoing.

An important caveat for analyses involving the tumor boundary in this study is that the tumor region was identified using the RGB volumes and not spatially co-registered histopathology. Thus, this study refers to the tumor boundary as the “RGB-defined” boundary. Although these images can provide bulk delineation between the tissue types, they do not provide gold-standard tumor identification on a microscopic level, which is particularly important near the tumor margin. In this region, understanding the agents’ behavior in the presence of infiltrative tumor cells and/or inflammatory cells is important.^27^^,^^28^ Ongoing studies in our lab are focused on recovering select 2D full brain sections during cryo-imaging for full-field histopathology, which would be registered to the imaged volume.

Further examination of the tumor boundaries at high resolution and in a larger sample size is a particular focus of our continued efforts to assess ALA-PpIX and SWIG. In addition, we are applying the same strategy to evaluate additional candidate agents, alone and in combination with one another. The evaluation strategy applied herein may serve as a model for rigorous 3D evaluation of FGS agents at high resolution in models close to human size.

## Supplementary Material





## Data Availability

All image data presented in this paper required to replicate these data are publicly available in figshare at https://doi.org/10.6084/m9.figshare.c.7221204.
