# Supplementary material for: Comparing spatial distributions of ALA-PpIX and indocyanine green in a whole pig brain glioma model using 3D fluorescence cryotomography
Source: J Biomed Opt. 2024 Sep 6;30(Suppl 1):S13704. doi: 10.1117/1.JBO.30.S1.S13704 (PMC11379406; doi:10.1117/1.JBO.30.S1.S13704)
Supplement: Supplementary file 1 [file JBO_030_S13704_SD001.pdf]

Supplemental Material

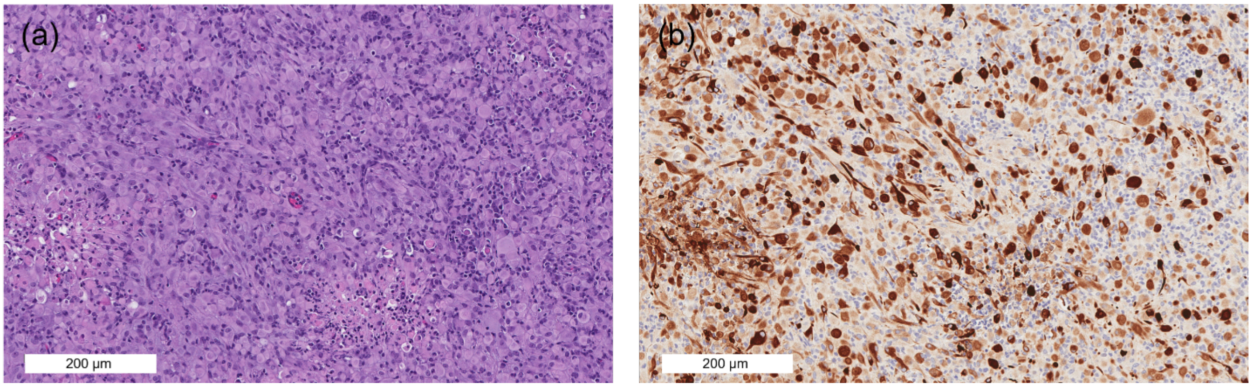

Fig. S1 Tumor pathology confirmation using hematoxylin and eosin (H&E) and glial fibrillary acid protein (GFAP) staining using an anti-GFAP primary antibody: (a - b) H&E and GFAP staining of tumor specimen at 16x respectively. Microscopic feature of this tumor include a disorganized proliferation of spindle shaped cells compatible with reactive astrocytes, small cells with dark nuclei, compatible with oligodendrocytes, and predominate population of round to oval cells with significant nuclear pleomorphism. The later population is compatible with a mixture of neoplastic glioma cells and glial macrophages and the primary tumor cell population was GFAP positive. Additional glioma tumor features include attempts at cell palisading and foci of necrosis demonstrating cell swelling/eosinophilia and nuclear pyknosis.

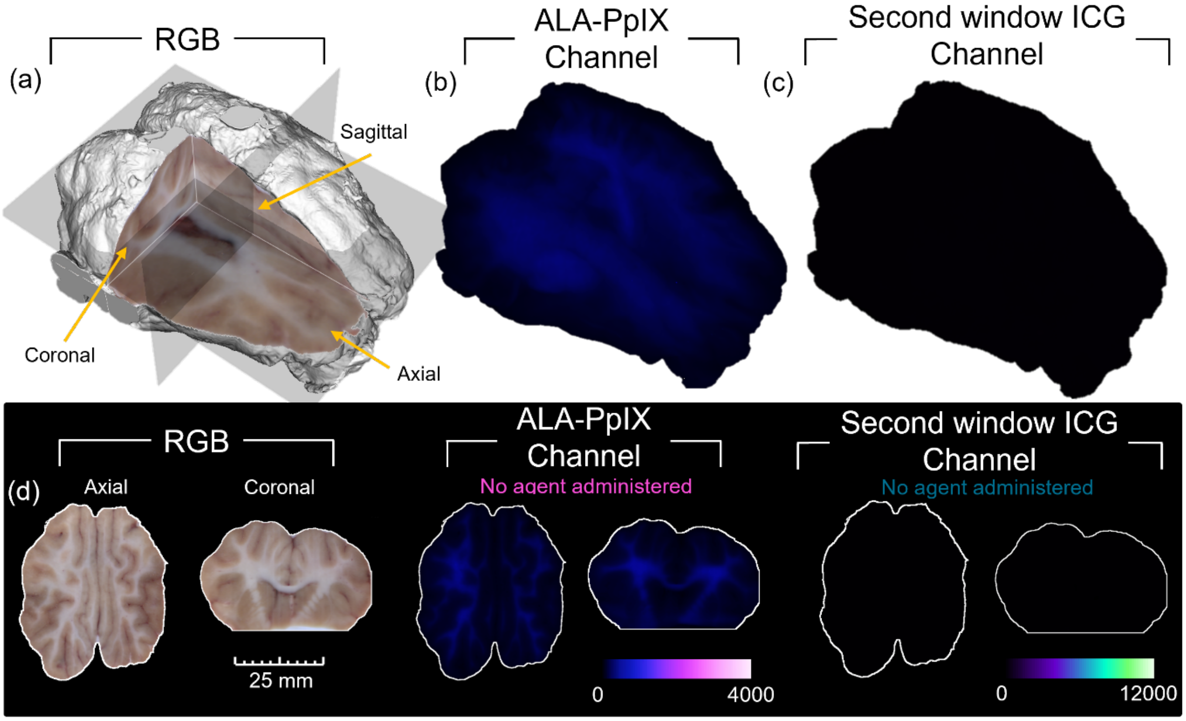

Fig. S2 Fluorescence cryo-imaging of a control pig brain without agent administration: (a) RGB rendering. (b) and (c) Maximum intensity projection (MIP) images of ALA-PpIX and ICG channels, respectively. (d) 2-D image slices for each channel sampled from the volume with the slice locations depicted by the two semitransparent planes shown in (a).
